# A Single-Cell Characterization of Human Post-implantation Embryos Cultured *In Vitro* Delineates Morphogenesis in Primary Syncytialization

**DOI:** 10.3389/fcell.2022.835445

**Published:** 2022-06-15

**Authors:** Yiming Wang, Xiangxiang Jiang, Lei Jia, Xulun Wu, Hao Wu, Yue Wang, Qian Li, Ruoxuan Yu, Hongmei Wang, Zhenyu Xiao, Xiaoyan Liang

**Affiliations:** ^1^State Key Laboratory of Stem Cell and Reproductive Biology, Institute of Zoology, Chinese Academy of Sciences, Beijing, China; ^2^University of Chinese Academy of Sciences, Beijing, China; ^3^Institute for Stem Cell and Regeneration, Chinese Academy of Sciences, Beijing, China; ^4^Beijing Institute for Stem Cell and Regenerative Medicine, Beijing, China; ^5^NHC Key Laboratory of Study on Abnormal Gametes and Reproductive Tract, Anhui Medical University, Hefei, China; ^6^Reproductive Medical Center, The Sixth Affiliated Hospital of Sun Yat-Sen University, Guangzhou, China; ^7^School of Life Science, Beijing Institute of Technology, Beijing, China

**Keywords:** single-cell RNA sequencing, human embryos, trophoblast differentiation, human trophoblast stem cells, cytoskeleton

## Abstract

Implantation of the human blastocyst is a milestone event in embryonic development. The trophoblast is the first cell lineage to differentiate during implantation. Failures in trophoblast differentiation during implantation are correlated to the defects of pregnancy and embryonic growth. However, many gaps remain in the knowledge of human embryonic development, especially regarding trophoblast morphogenesis and function. Herein, we performed single-cell RNA sequencing (scRNA-seq) analysis on human post-implantation embryos cultured *in vitro*. A hierarchical model was established, which was characterized by the sequential development of two primitive cytotrophoblast cell (pCTB) subtypes, two primitive syncytiotrophoblast subtypes, and migrative trophoblast cells (MTB) after the trophectoderm . Further analysis characterized cytoskeleton transition of trophoblast cells and morphogenesis, such as irregular nuclei, cell cycle arrest, and cellular aging during implantation. Moreover, we found syncytialization of hTSCs could mimic the morphogenesis, serving as a powerful tool for further understanding of the mechanism during the implantation stage of pregnancy. Our work allows for the reconstruction of trophoblast cell transcriptional transition and morphogenesis during implantation and provides a valuable resource to study pathologies in early pregnancy, such as recurrent implantation failure.

## Introduction

The first connection between the mother and the fetus, through the progression of embryo implantation, is essential for a successful mammalian pregnancy. Compared with other mammalian species, human reproduction is surprisingly inefficient (Edwards et al., 1969). Only about 30% of natural conceptions lead to a successful pregnancy in human (Wang et al., 2003; Zinaman et al., 2019). Among the pregnancies that are lost, implantation defects contribute to approximately 85% of these pregnancy failures (Macklon et al., 2002; Zinaman et al., 2019). *In vitro* fertilization (IVF) treatments could help to overcome the problems described above. However, despite the advances in assisted reproductive technologies, the majority of IVF procedures are not effective mainly as a result of implantation failure. Implantation defects that compromise embryonic development account for 48.6% of the total pregnancy failures in assisted reproduction technology (Wang et al., 2003; Boomsma et al., 2009; Koot et al., 2012). All the statistics on both natural pregnancy and IVF indicate that implantation is a critical step for human reproduction. However, the regulatory mechanisms underlying human implantation are largely unexplored.

The success of implantation is dependent on a competent blastocyst, receptive endometrium, and successful cross-talk between the embryonic and maternal interfaces. Implantation failure can result from the low quantity of the embryos, poor uterine receptivity (Palomba et al., 2021), and endocrine milieu (Baird et al., 1991), which due in part to embryo and endometrial synchrony. Low-quantity embryos with whole chromosome aneuploidy and deficient ICM and trophoblast differentiation have negligible implantation potential (Tiegs et al., 2021). Trophoblast cells play important roles in mediating the interaction between the fetus and mother during implantation (Enders and Schlafke 1969). Human trophoblast cells originate from an outer layer of the blastocyst, namely trophectoderm (TE), covering the pluripotent inner cell mass (ICM) (Hertig et al., 1956). Moreover, human embryo implantation is accompanied by a series of morphogenesis, such as the generation of a bilaminar disc, formation of a pro-amniotic cavity, appearance of the prospective yolk sac, and trophoblast differentiation (Deglincerti et al., 2016; Shahbazi et al., 2016). It was revealed that the exit of epiblasts from naive pluripotency in cultured human post-implantation embryos triggers amniotic cavity formation and developmental progression (Shahbazi et al., 2017). However, to date, the morphogenesis in trophoblast cells during implantation has not been analyzed in detail. To implant the maternal uterus, TE will differentiate into multinuclear primitive syncytiotrophoblast (pSTB) via a process named primary syncytialization. Our knowledge about human trophoblast development during implantation is largely gleaned from the description of anatomical structures from early implantation sites present in hysterectomy specimens of the Boyd collections (Centre of Trophoblast Research, Cambridge) and the Carnegie collections (Human Developmental Anatomy Center, Washington DC). According to the Carnegie collections, pSTB forms at around day 8 p.f. (post fertilization). Moreover, clusters of large nuclei are contained in some of the syncytial masses (Hertig et al., 1956; Enders and Schlafke 1969). Small vacuoles appear within the pSTB at the implantation pole. The vacuoles enlarge rapidly and become confluent, forming a system of lacunae. Subsequently, lacunae spread over the whole surface of the trophoblast cells (Hertig et al., 1956). Therefore, the noticeable feature of primary syncytialization is the increase in nuclear volume and lumenogenesis. However, owing to the scarcity of access to peri-implantation or post-implantation human embryos *in vivo*, the morphologies, detailed gene expression, and regulatory network dynamics underlying trophoblast lineage differentiation during the peri-implantation stage remain to be elucidated.

The combination of the human embryo *in vitro* culture to the post-implantation stage and single-cell RNA sequencing (scRNA-seq) technology has revealed that populations of early trophoblast cells are heterogeneous (Lv et al., 2019; West et al., 2019; Xiang et al., 2020). For instance, Lv et al. (2019) divided early trophoblast cells from day 6 to day 10 into six subpopulations. West et al. (2019) divided early trophoblasts into CTB, pre-STB, STB, pre-MTB (pre-migrative trophoblast cells), and MTB from day 8 to day 12 and revealed that IFN signaling increased as development proceeded. It has also been shown that early EVTs can secrete hormones (Xiang et al., 2020). All studies are focused on specific cell types or gene functions but do not elucidate key developmental routes in trophoblast specification. This highlights the need for systematic transcriptomic studies of the pre-implantation and post-implantation human trophoblast cells.

Combining the *in vitro* culture of human embryos to the post-implantation stage with scRNA-seq technology, we developed a comprehensive resource characterizing the transcriptional dynamics of trophoblast specification. We integrated our human scRNA-seq datasets with published interval human datasets and unraveled novel sequential early trophoblast development during implantation. We identified the cytoskeleton transition of trophoblast cells and morphogenesis, such as irregular nuclei, cell cycle arrest, and cellular aging during implantation. In summary, our study expands our knowledge of human trophoblast cell development and function during implantation.

## Materials and Methods

### Ethics Statement

This work was approved by the Ethics Committee of the Center for Reproductive Medicine, Sixth Affiliated Hospital of Sun Yat-Sen University (Research license 2019SZZX-008). The Medicine Ethics Committee of the Center for Reproductive Medicine, Sixth Affiliated Hospital of Sun Yat-Sen University is composed of 11 members, including experts in laws and science and clinicians with relevant expertise. The Committee evaluated the scientific merit and ethical justification of this study and conducted a full review of the donation and use of these samples.

The informed consent process for embryo donation complied with the International Society for Stem Cell Research (ISSCR) Guidelines for Stem Cell Research and Clinical Translation (2021) and the Ethical Guidelines for Human Embryonic Stem Cell Research (2003) jointly issued by the Ministry of Science and Technology and the Ministry of Health of People’s Republic of China. The ethical and regulatory framework set forth by the Center for Reproductive Medicine, Sixth Affiliated Hospital of Sun Yat-Sen University, clearly specified that informed consent could only be obtained if eligible participants were provided with all necessary information about the study and had an opportunity to receive proper counseling. The consent form clearly described the goals and related clinical procedures for the study. No financial inducements were offered for the donations.

All donated embryos in this study were obtained from frozen embryos from couples who had already signed informed consent. The study employed standard clinical protocols for embryo collection, cryopreservation, thawing, and culture procedures. The human embryos used in this work were obtained at 5 or 6 days postfertilization (d.p.f.). Cryopreserved embryos were thawed using the Kitazato Thawing Media Kit VT802 (Kitazato Dibimed) depending on the protocol used for freezing according to the manufacturer’s instructions. The embryos were cultured in the single-step embryo culture medium (LifeGlobal) covered with oil (LifeGlobal). Embryos with normal morphology were utilized in this study.

### Culture of Human Trophoblast Stem Cells

Culture of human trophoblast stem cells (hTSCs) from human embryos was performed as previously described (Okae et al., 2018). hTSCs were cultured in hTSCs medium (DMEM/F12 supplemented with 0.1 mM 2-mercaptoethanol, 0.2% FBS, 0.5% Penicillin-Streptomycin, 0.3% BSA, 1% ITS-X supplement, 1.5 μg/ml l-ascorbic acid, 50 ng/ml EGF, 2 μM CHIR99021, 0.5 μM A83-01, 1 μM SB431542, 0.8 mM VPA and 5 μM Y27632). hTSCs were routinely passaged every 3 days at a 1:4–1:6 ratio. hTSCs were dissociated with TrypLE for 8 min at 37°C, and the single cells were passaged to a new Collagen I-coated 4-well plate and cultured in hTSCs medium. hTSCs were grown to 80% confluence in the hTSCs medium and dissociated with TrypLE for 8 min at 37°C. For the induction of STB, hTSCs were seeded in a μ-Slide 8-well dish (ibidi, 80826) pre-coated with 2.5 μg/ml Collagen I at a density of 1 × 10^4^ cells per well and cultured in 200 μl of STB medium (DMEM/F12 supplemented with 0.1 mM 2-mercaptoethanol, 0.5% Penicillin-Streptomycin, 0.3% BSA, 1% ITS-X supplement, 2.5 μM Y27632, 2 μM forskolin, and 4% KSR). The medium was replaced every 2 days, and the cells were immunostained on day 6.

### Human Embryo Culture

The human embryo culture procedure was performed as previously described (Deglincerti et al., 2016; Shahbazi et al., 2016). Briefly, human blastocysts (day 5 and day 6) were thawed with the Kitazato Thawing Media Kit VT802 (Kitazato Dibimed), and the embryos were exposed to acidic Tyrode’s solution (Sigma) to remove the zona pellucida. The zona pellucida-free embryos were cultured in an 8-well plate (ibidi, 80826) containing warmed *in vitro* culture medium 1 (IVC1 medium, 200 µl per well). The embryos were usually attached to the bottom of the plate after 48 h, and then half of the IVC1 medium was replaced by *in vitro* culture medium 2 (IVC2 medium). The culture medium was exchanged with IVC2 medium every 24 h.

### Immunofluorescence of Human Embryos

Embryos at different time points were first washed with sterile PBS at least three times. Then, the embryos were fixed with the fixative solution (4% PFA in PBS) at room temperature for 30 min. The embryos were washed three times with washing solution (0.1% vol/vol Tween 20 in PBS) at room temperature for 10 min each time. The embryos were incubated in permeabilization solution (0.5% vol/vol Triton X-100 in PBS) at room temperature for 30 min and washed three times in washing solution. Then, the embryos were incubated in a blocking solution (3% BSA in washing solution) at room temperature for at least 1 h before adding primary antibodies diluted to 1:200/1:100 in blocking solution at 4°C overnight. The embryos were washed three times in washing solution at room temperature for 10 min. The embryos were incubated with fluorescence-conjugated secondary antibodies (donkey anti-mouse AlexaFluor® 568, Thermo Fisher Scientific, A10037; donkey anti-rabbit AlexaFluor® 647, Thermo Fisher Scientific, A31573; and AlexaFluor® 488 Phalloidin, Thermo Fisher Scientific, A12379) and DAPI (diluted 1:200) in blocking solution at room temperature for 1 h. The embryos were washed twice in the washing solution at room temperature for 10 min. Finally, the embryos were observed using a Zeiss LSM 780 or 880 confocal microscope (Zeiss, Jena, Thuringia, Germany). All immunofluorescence images were processed with Imaris software. Note that all the staining procedures were performed carefully under a stereomicroscope.

### Single Cell Collection

Single cells were isolated from embryos at embryonic days 8, 9, 10, 11, 12, and 13, incubated with TrypLE Express reagent for 10–15 min at 37°C, and dissociated into single cells. Single cells were randomly picked with a mouth pipette in 0.1% BSA and then transferred to 2.5 μl lysis buffer. The cells were transferred into a 0.2 ml PCR tube (Eppendorf) containing cell lysis buffer and kept at −80°C for library preparation.

### Construction of the Single-Cell RNA-Seq Library

We used a modified Smart-seq2 protocol to construct the single-cell RNA-seq library (Picelli et al., 2014). In short, the cells were lysed to release all RNAs, then, the mRNAs were captured with barcoded oligo-dT primers with an anchor sequence and unique molecular identifier (UMI) sequences. The mRNAs were reverse-transcribed to first-strand cDNAs. Then, the preamplification step was performed to increase cDNA yields. Finally, cDNAs from different cells were pooled together with different barcodes. After five cycles of PCR, the index sequence with biotin modification was added at the 3′ ends of the cDNAs. Following DNA fragmentation with an ultrasonicator, we used Dynabeads C1 (65002, Invitrogen) to enrich the 3′ cDNAs to construct the library with the Kapa Hyper Prep Kit (KK8505, Kapa Biosystems). Libraries were then sent to Novogene for quality control and sequencing. The size distribution was analyzed on an Agilent 2100 bioanalyzer, and a 300–600 bp range peak was observed in qualified libraries, and the concentrations of qualified libraries were more than 10 ng/μl. The qualified libraries were sequenced on the Illumina Hiseq4000 platform with 150-bp paired-end reads. Every library contains 96 transcriptomes of cells. And 10 libraries were sequenced and multiplexed together in a lane. Finally, at least 400,000 reads were sequenced for each cell. The qualified libraries were sequenced on the Illumina HiSeq XTEN platform using the 150 bp paired-end reads (PE150) strategy.

### Read Mapping and Gene Expression Quantification

The paired-end reads of Smart-seq2 data were processed using the custom scripts of Drop-seq_tools-2.0.0. For read 2, bases 1 to 8 were tagged with cell-barcode “XC”, and bases 9 to 16 were tagged with UMI “XM”. After removing the adaptors and TSO sequences and poly(A) sequences, STAR aligner was used to align the filtered reads to the human hg38 reference genome, and reads were annotated with the GRCh38.84 annotation file. A gene expression matrix (count value) was generated with the “DigitalExpression” command function. The raw data and processed gene expression matrix data were deposited in the NCBI Gene Expression Omnibus (GEO) database with the accession GSE156456. The Smart-seq2 data from GSE109555 were also processed using the above method.

### Visualization and Clustering of the Single-Cell Data

We mainly used the Seurat3 R package to analyze the Smart-seq2 single-cell data (Butler et al., 2018; Stuart et al., 2019). A Smart-seq2 count matrix was used to create the Seurat object. Only genes expressed in more than three cells were retained. Regarding the cells that were sequenced, only cells with a percentage of mitochondrial genes less than 15% expressed in more than 2,000 genes were retained (1,014 cells). For cells from GSE109555, we filtered out cells with a percentage of mitochondrial genes greater than 4% and cells that expressed less than 8,000 genes, and 3,859 cells were retained for subsequent analysis. After the normalization step, we computed highly variable genes with the “mean.var. plot” method. Following scaling all genes in the data, we performed linear dimensional reduction with highly variable genes by default. The “ElbowPlot” function was chosen to determine the dimensionality to perform nonlinear dimensional reduction (UMAP). The graph-based clustering approach was used to cluster the cells by the “FindNeighbors” and “FindClusters” functions. Single-cell data were visualized by the “Dimplot” function. “FeaturePlot”, “VlnPlot” and “DoHeatmap” functions were used to display the gene expression levels.

### DEG and GO Analysis

Highly expressed genes of each cell cluster were analyzed using the Seurat “FindAllMarkers” function on the log-transformed expression matrix. Differentially expressed genes between two cell clusters were found using the Seurat “FindMarkers” function. GO biological process analysis was performed via the clusterProfiler R package.

### Pseudotime Analysis

The Monocle2 R package was used to perform the pseudotime analysis (Trapnell et al., 2014; Qiu et al., 2017). Briefly, the New Seurat2 object was built with a count value of 3,859 cells, and only those genes that were expressed in more than three cells were retained. The Seurat2 object was then converted to the Monocle2 object by the “importCDS” function of Monocle2. The highly variable genes computed by Seurat3 were used to perform the unsupervised ordering of the cells. The “plot_cell_trajectory” function was used to plot pseudotime images, and cells were colored using the metadata that Seurat3 used.

### SCENIC Analysis

SCENIC analysis was carried out following the SCENIC command line protocol (Aibar et al., 2017). The SCENIC command line version was used to perform gene regulatory network inference, regulon prediction and cellular enrichment (AUCell) processes with the count data and metadata of 4,873 cells. SCENIC AUC UMAP was generated using the AUC matrix. Regulon specificity scores (RSS) were computed based on the cell clusters identified by Seurat. We chose the top 10 regulons for each cell cluster. Finally, the AUC heat map was plotted by the pheatmap R package, and only genes were clustered.

### Analysis of Hormone Genes

Hormone genes were collected from published reports (Guibourdenche et al., 2009; Liu et al., 2018). The scaled data from the Seurat3 object were used to plot the heatmap with the pheatmap R package. The cells were arranged according to the cell clusters computed by Seurat3, and genes were clustered by the default method of the pheatmap package.

### Statistical Analysis

All images were acquired by the ZEISS LSM 780 or ZEISS LSM 880 confocal laser-scanning microscopes, with the ×20 air objectives, ×40, and ×63 oil-immersion objectives. Images were acquired with 1 μm Z separation. Three-dimensional visualizations were performed using Imaris. All analyses were carried out using open-source image analysis software including Zeiss LSM Image Browser Software, Imaris Software, and Fiji Image J (NIH). Results were shown as mean ± SEM. Statistical parameters including statistical analysis and statistical significance reported in the figure legends and supplementary figure legends were obtained using a *t*-test through GraphPad Prism8. Significance was defined as *p < 0.05; **p < 0.01; ***p < 0.001.

## Results

### Single-Cell RNA Sequencing of Post-Implanted Human Embryos From Day 8 to Day 13 Post Fertilization

**FIGURE 1:**
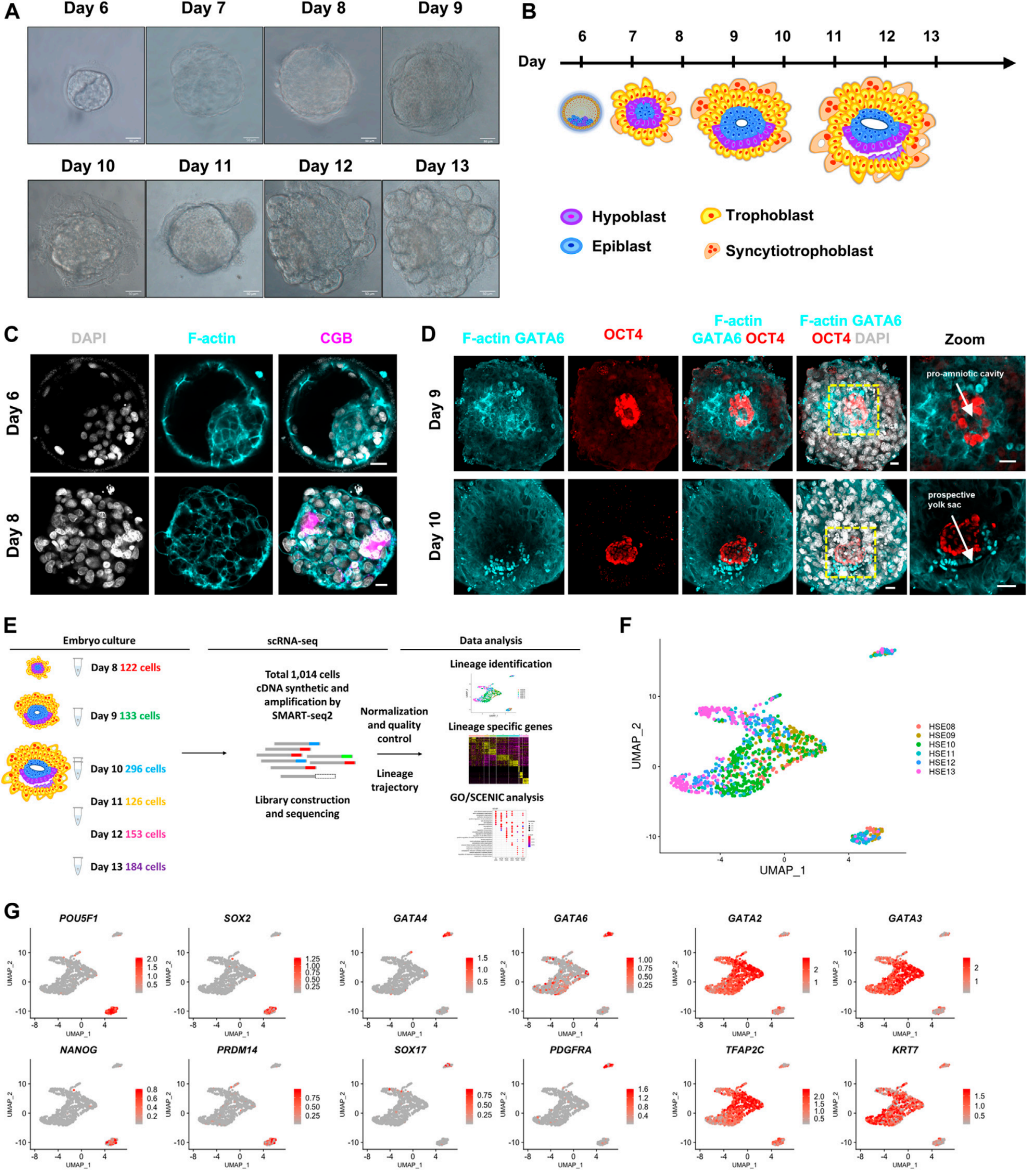
Global expression profiling of human embryo cells using single-cell RNA-seq and cell type identification. **(A)** Bright-field images of *in vitro* cultured human embryos from day 6 to day 13. Scale bars, 50 μm. **(B)** Cartoons of human embryo implantation morphogenesis based on Carnegie series. **(C)** Immunostaining of day 6 and day 8 human embryos (*n* = 3 and *n* = 3) for F-actin and CGB. DAPI, grey, DNA. Scale bars, 20 μm. **(D)** Immunofluorescence of day 9 and day 10 human embryos (*n* = 3 and *n* = 1) for F-actin, GATA6, and OCT4. Images showing the pro-amniotic cavity and prospective yolk sac marked by white arrows. DAPI, grey, DNA. Scale bars, 20 μm. **(E)** Workflow depicting the strategy of derivation of single cell from post-implantation human embryos. Number of cells every embryonic day (day 8-day 13) retained after quality filtering were shown. **(F)** UMAP plots showing single-cell transcriptomes of human embryos from day 8–day 13. **(G)** UMAP plots showing the expression patterns of the lineage markers in human embryos. Color key from grey to red indicates relative expression levels from low to high, respectively.

Following implantation, the human embryo undergoes major morphogenetic transformations that establish the future body plan, for example, the formation of a pro-amniotic cavity, prospective yolk sac, and the diversification of trophoblasts (Luckett 1975; Deglincerti et al., 2016; Shahbazi et al., 2016). To recapitulate the developmental processes of the early trophoblast development in post-implantation human embryos, we cultured blastocysts donated from patients undergoing *in vitro* fertilization (IVF) treatment through a similar *in vitro* culture condition as described previously (Bedzhov et al., 2014; Deglincerti et al., 2016; Shahbazi et al., 2016). Donated human embryos were thawed and cultured in 8-well chambers from day 8 post fertilization until day 13 post fertilization (Figures 1A,B). To determine the morphogenetic change of *in vitro* cultured embryos, we fixed and immunostained human embryos at different culture time points. By day 8, the embryos attached to the dishes and the primitive syncytium formed at this stage, which was identified by the expression of human chorionic gonadotropin subunit beta (CGB) (Figure 1C). By day 9, the embryos completely collapsed and acquired a lumen at the center of OCT4-expressing cells, indicating the formation of the pro-amniotic cavity within the epiblast (Figure 1D). By day 10, the embryos formed a second cavity. The cavity immediately below the epiblast was surrounded by GATA6-expressing cells, indicating the formation of the prospective pro-amniotic cavity (Figure 1D). At the same time, we found that human embryos cultured *in vitro* possessed columnar-shaped and squamous-shaped OCT4-expressing amniotic epithelium cells, indicating the formation of bilaminar discs (Figure 1D). The emergence of the primitive syncytium, prospective pro-amniotic cavity, primary yolk sac, and bilaminar disc suggested that we could remodel major morphogenesis events of post-implantation human embryos *in vitro*.

To determine the temporal gene expression profile underlying this critical morphogenesis of the human embryos beyond implantation, we generated scRNA-seq profiles from a total of 12 human embryos at six time points (at 1 day interval from day 8 to day 13) using a modified Smart-seq2 protocol. Single-cell suspensions were prepared as previously described (Zhou et al., 2019), and scRNA-seq analysis was performed. After a strict quality control test, we acquired single-cell transcriptomes of a total of 1,014 cells from six indicated time points (Figures 1E,F; Supplementary Figures 1A,B). We then generated expression cluster-specific marker genes by performing differential gene expression analysis to define each cluster. We defined three distinct clusters based on the expression of known markers together with the most significant enriched genes in each cluster. *POU5F1* (known as *OCT4*), *SOX2*, *NANOG*, and *PRDM14* for epiblast cells, *GATA6*, *GATA4*, *SOX17*, and *PDGFRA* for primitive endoderm cells, and *GATA3*, *GATA2*, *TFAP2C*, and *KRT7* for trophoblast cells (Figure 1G). Together, our clustering algorithms of the transcriptome data showed stepwise diversification of the epiblast, primitive endoderm, and trophoblast lineages.

### Trophoblast Differentiation From Pre- to Post-Implantation

**FIGURE 2:**
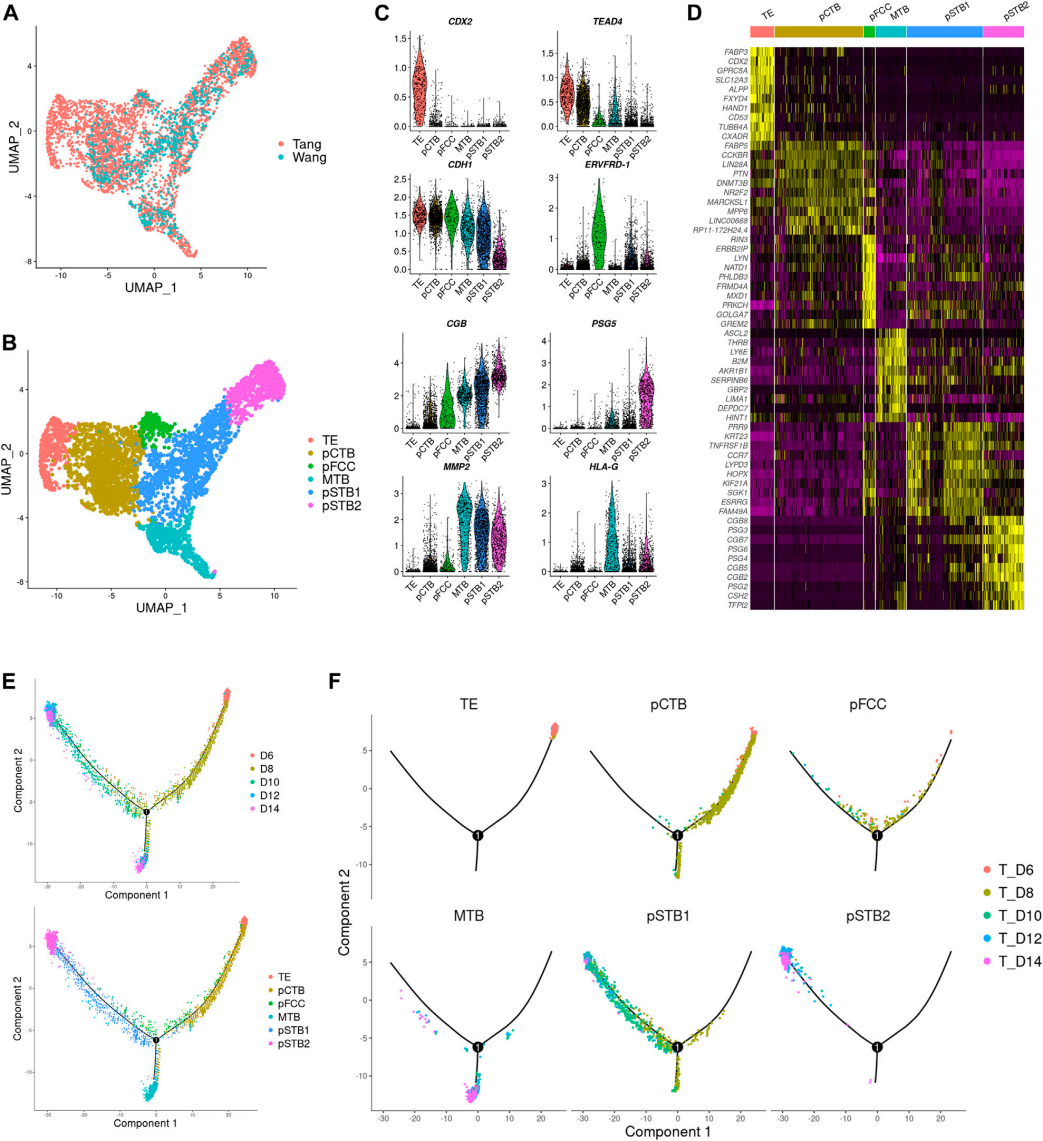
Distinct subtypes in early trophoblast cells during implantation. **(A)** Joint visualization of our datasets together with published datasets. Tang represents published datasets; Wang represents our datasets. **(B)** UMAP plots showing the expression patterns of early trophoblast cells in different clusters. TE, trophectoderm; pCTB, primitive cytotrophoblast; pFCC, primitive fusion-competent trophoblast cells; MTB, migrative trophoblast cells, pSTB1, primitive syncytiotrophoblast 1; pSTB2, primitive syncytiotrophoblast 2. **(C)** Violin plots showing the markers expression of different clusters. **(D)** Heat map showing representative DEGs in different clusters. Color key from purple to yellow indicates relative expression levels from low to high, respectively. **(E,F)** Pseudotime analysis was assigned to each cell showing embryonic day and lineage assignment, with cells colored by embryonic day (upper) and cell clusters (lower). Three-dimensional diffusion map representation of all cells at different culture time points, showing lineage assignment on each embryonic day **(F)**.

Trophoblast differentiation occurs in humans as the blastocyst implants into the uterus during peri-implantation. To obtain a better understanding of trophoblast differentiation in the context of trophoblast cell subtypes and their unique transcriptional signatures in the human embryos in culture, we integrated previously published single-cell expression profiles of human embryos cultured *in vitro* at peri-implantation stages, including the pre-implantation stage (which contained samples of day 6, 8, 10, 12, and 14) (Zhou et al., 2019) with our post-implantation transcriptome sequential datasets (which contained samples of days 8, 9, 10, 11, 12, and 13). Integration analysis revealed a high cluster similarity between the datasets and confirmed the identification of the major cell types present in post-implantation human embryos, suggesting that the single-cell samples of 1 day interval and 2 days interval developmental times had similar global gene expression patterns (Figure 2A).

Next, we carried out a dimensionality reduction algorithm using the most variable genes across all trophoblast cells in scRNA-seq gene expression data. Six distinct cell types of trophoblast cells were identified as shown in Figure 2B. We generated expression cluster-specific marker genes by performing differential expression gene (DEG) and used well-known cell type markers to identify cell clusters. Namely *CDX2*, mouse trophectoderm gene, for TE (Cluster 1). *CDH1* and *TEAD4*, high expressions in cytotrophoblast cells, for pCTB (Cluster 2). *ERVFRD-1*, a molecule induced cell fusion, for primitive fusion-competent cells (pFCC) (Cluster 3). *MMP2*, a member of the matrix metalloproteinase family, for migrative trophoblast cells (MTB) (Cluster 4). *CGB*, a marker of hormone activity, for pSTB1 and pSTB2 respectively (Cluster 5 and Cluster 6), as shown in Figures 2B,C. The DEGs between these subtypes were shown in Figure 2D. To further reconstruct the continuous differentiation trajectory of the newly annotated trophoblast cell clusters, we assigned the pseudotime assay to each cell cluster (Figure 2E). Pseudotime analysis was carried out according to cell lineages and developmental time (Figure 2F), which indicated segregation from TE to pCTB and pSTB on day 8 as well as segregation from pCTB to pSTB and MTB cells on day 10. The first segregation from TE to pCTB and pSTB emerged on day 8, which coincided with the time when the embryos attached to the dishes, in agreement with the previous reports of human embryos indicating that human trophoblasts arise from the trophectoderm (TE) once the blastocyst implanted into the uterus. We also immunostained day 6, day 8, and day 10 human embryos for GATA3 and CGB to identify TE, pCTB, and pSTB (Supplementary Figures 2A–C). Taken together, our results identified subtypes of human trophoblast cells and revealed a linear trajectory of trophoblast cell subtypes during implantation based on scRNA-seq datasets.

### Nuclear Enlargement and Deformation Are Required for Primitive Syncytiotrophoblast Development

**FIGURE 3:**
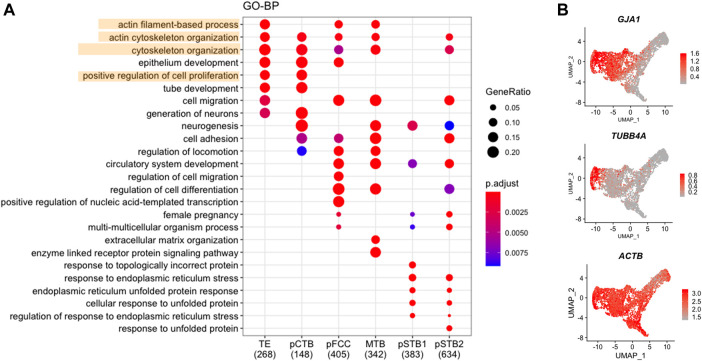
Cytoskeleton-associated genes decreased in primitive syncytiotrophoblast. **(A)** Dot plots showing enriched GO terms and *p*-value of distinct clusters of trophoblast cells. **(B)** UMAP plots showing the expression levels of representative genes for trophoblast cells. Color key from grey to red indicates relative expression levels from low to high, respectively.

**FIGURE 4:**
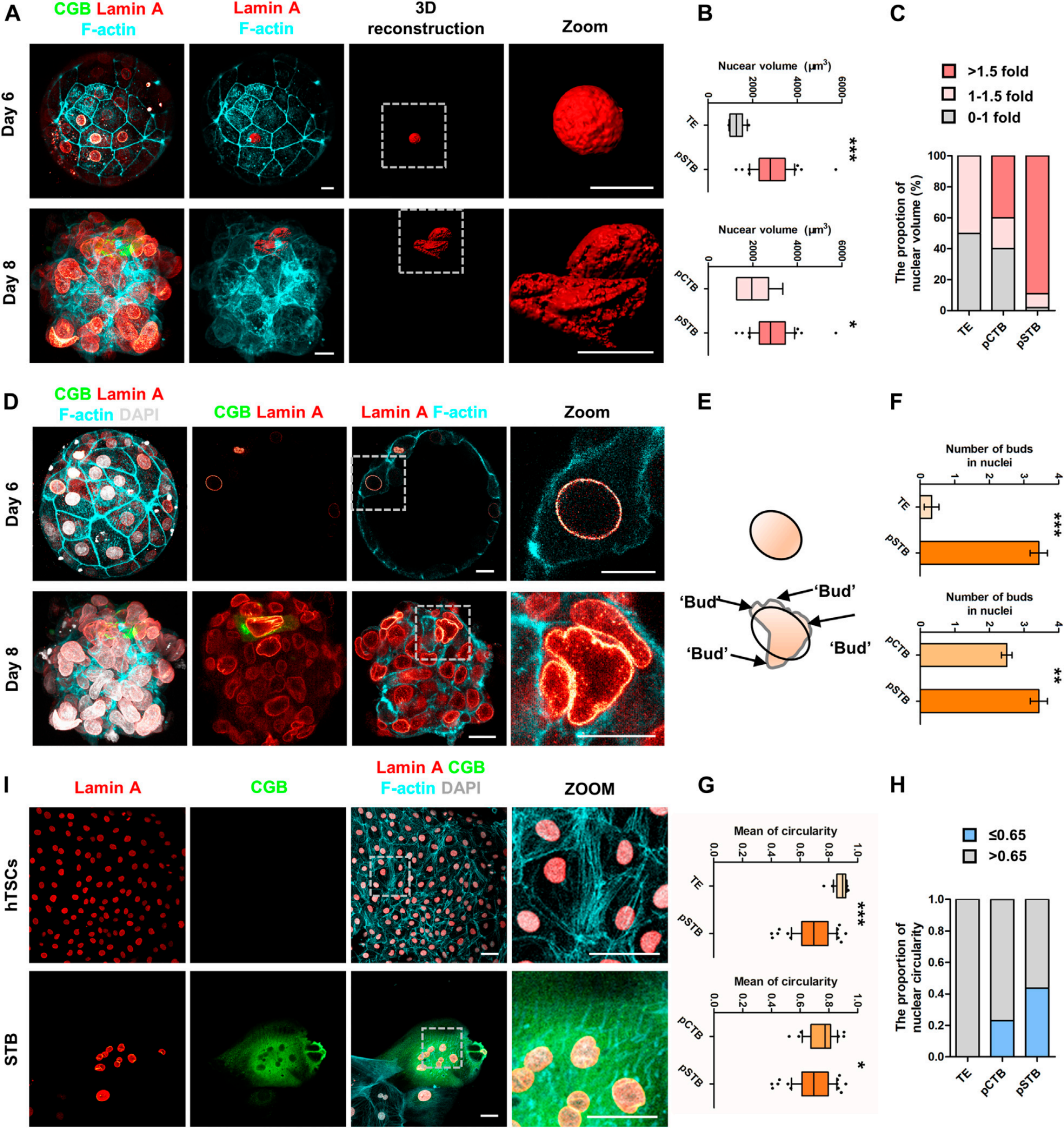
Nuclear enlargement and deformation in primitive syncytiotrophoblast. **(A)** Immunostaining of day 6 and day 8 human embryos (*n* = 3 and *n* = 3) cultured *in vitro* for CGB, Lamin A, and F-actin. 3D reconstruction of nuclei basing on the immunostaining results (shown in red). Dotted rectangle represented regions in the embryos that are shown with higher magnification. Scale bars, 20 μm. **(B)** Quantification of nuclear volume in TE, pCTB, and pSTB of human embryos. *n* = 3 human embryos per group. Data are shown as mean ± SEM. Unpaired two-tailed Student’s *t*-test, *p < 0.05; ***p < 0.001. **(C)** Bar graph showing the proportions of nuclear volume in TE, pCTB, and pSTB of human embryos. **(D)** Immunostaining of day 6 and day 8 human embryos (*n* = 3 and *n* = 3) cultured *in vitro* for CGB, F-actin, and Lamin A. Dotted rectangle represented regions in the embryos that are shown with higher magnification. DAPI, grey, DNA. Scale bars, 20 μm. **(E)** Cartoons of nuclear morphology based on immunostaining results. Protrusions are the projecting point after Elliptical Fourier Analysis. **(F)** Quantification of protrusion numbers in **(D)**. Data are shown as mean ± SEM. *n* = 3 human embryos per group. Unpaired two-tailed Student’s *t*-test, **p < 0.01; ***p < 0.001. **(G)** Quantification of circularity in TE, pCTB, and pSTB of human embryos. *n* = 3 human embryos per group. Data are shown as mean ± SEM. Unpaired two-tailed Student’s *t*-test, *p < 0.05; ***p < 0.001. **(H)** Bar graph showing the proportions of nuclear circularity. **(I)** Immunostaining of hTSCs and STB for Lamin A, CGB, and F-actin. Dotted rectangle represented regions that are shown with higher magnification. DAPI, grey, DNA. Scale bars, 50 μm.

To further characterize the molecular characteristics of each subpopulation involved in implantation, Gene Ontology (GO) analysis was performed for the DEGs that were specifically expressed at high levels in each trophoblast subpopulation from pre-implantation to post-implantation (Figure 3A). It was shown that TE-specific genes were enriched for the “cytoskeleton organization”. Dynamic cytoskeletal networks are required for correct trophoblast lineage specification following implantation. For further mechanistic insights, we tested whether the cytoskeleton-related genes could potentially mediate TE specification. We found that the cytoskeleton-associated genes *GJA1* and *TUBB4A* were significantly downregulated during trophoblast differentiation, as shown in Figure 3B. Interestingly, we found the expression of *ACTB* also decreased during implantation (Figure 3B). As one of the major components of the cytoskeleton, actin filaments build the physical basis to fulfill their cellular function. Actin filaments were reported previously as regulators of nuclei morphology (Kim et al., 2017). However, little is known about the molecular mechanism underlying actin filaments dynamics during syncytialization. Moreover, in dendritic cells, actin-based mechanisms facilitate nuclear deformation (Thiam et al., 2016). To determine whether the morphogenesis of nuclei had been changed during implantation in different trophoblast clusters in our culture system, we cultured human embryos and immunostained day 6 (pre-implantation) and day 8 (post-implantation) human embryos for lamin A, F-actin, and CGB (a marker of pSTB) followed by 3D reconstruction of the nuclei. Similarly, immunofluorescence (IF) analyses of day 6 human embryos revealed that the shape of TE is oval, whereas the nuclei in trophoblast cells of day 8 human embryos exhibited nuclear malformations. CGB^+^ pSTB had a significantly increased fraction of abnormally shaped nuclei (Figures 4D,E). We found the nuclei in pSTB of human embryos were considerably enlarged when compared with those in TE and cytotrophoblast cells (Figures 4A,B), which is similar to the human embryos *in vivo*. The majority of the nuclei in pSTB (89%) were found larger than nuclei from mononucleated trophoblast cells (Figure 4C).

Subsequently, we calculated typical examples of nuclear shape alterations, specifically irregular nuclei (Cao et al., 2011; Takaki et al., 2017). The results showed more irregular nuclei in pSTB compared with the ones in TE and pCTB cells (Figure 4F). To more quantitatively assess the degree of irregular nuclear shape, we computed the nuclear circularity (4π × area perimeter^−2^) of TE cells, pCTB cells, and pSTB. For a circular shape, the nuclear circularity had a value of 1; less nuclear roundness was associated with smaller values, and an altered nuclear envelope was defined as circularity ≤ 0.65 (Lammerding et al., 2005; Barascu et al., 2012). Indeed, the nuclei in pSTB significantly decreased the mean circularity and increased the percentage of abnormally shaped nuclei (circularity ≤ 0.65) compared with TE cells and pCTB cells (Figures 4G,H). These results demonstrated that primitive syncytiotrophoblast possesses irregular nuclei, such as enlargement and deformation.

The above results revealed irregular nuclei are the main feature of trophoblast morphogenesis after implantation. Given the ethical restrictions and the limited number of human embryos available for loss/gain-of-function studies, we turned to derive hTSCs as described from human blastocysts to remodel trophoblast morphogenesis during implantation. To determine whether hTSCs and STB differentiated from hTSCs mimic irregular nuclei, we cultured hTSCs in hTSCs medium and STB medium for 5 days, respectively, and performed IF analysis with Lamin A, CGB, and F-actin antibodies. We found that CGB^+^ STB exhibited obvious nuclear malformations compared with hTSCs (Figure 4I). Taken together, these results demonstrated nuclei were enlarged and malformed in primitive syncytiotrophoblast of human embryos and STB differentiated from hTSCs.

### Loss of “Cytoplasmic Bridge” in Primitive Syncytiotrophoblast

**FIGURE 5:**
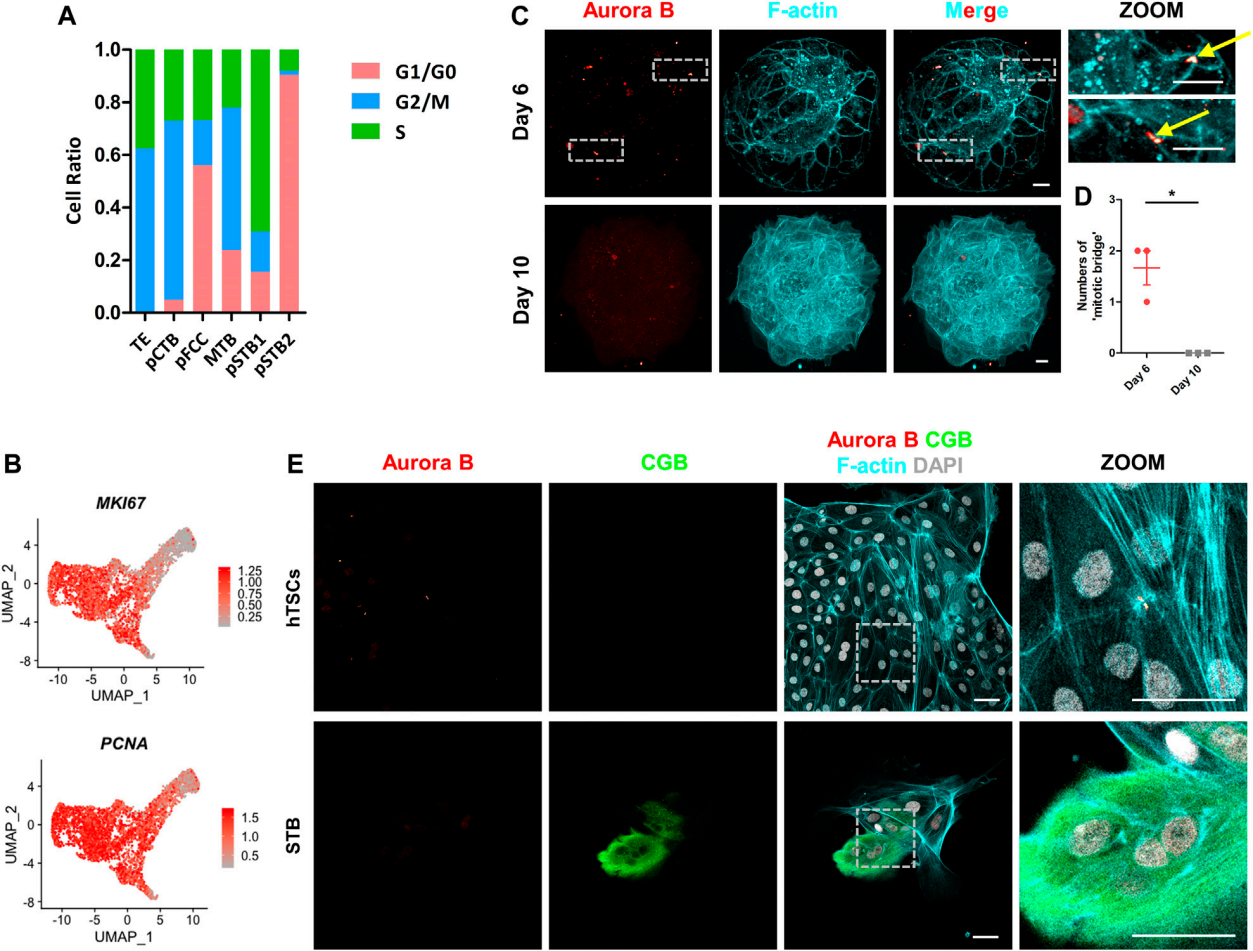
Loss of “cytoplasmic bridge” in primitive syncytiotrophoblast. **(A)** Bar graph showing the ratios of cells in different phases to total cells in each cluster according to the expression of S- and G2/M-phase genes. G1/G0-phases are the cells not in the S or G2/M phases. **(B)** UMAP plots showing the indicated genes in trophoblast cells. Color key from grey to red indicates relative expression levels from low to high, respectively. **(C)** Immunostaining of day 6 and day 10 human embryos (*n* = 3 and *n* = 3) cultured *in vitro* for F-actin and Aurora B. Dotted rectangle represented regions that are shown with higher magnification. Yellow arrows indicate “cytoplasmic bridges”. Scale bars, 20 μm. **(D)** Quantification of “cytoplasmic bridge” number in **(C)**. Data are shown as mean ± SEM. *n* = 3 human embryos per group. Unpaired two-tailed Student’s *t*-test, *p < 0.05. **(E)** Immunostaining of hTSCs and STB for Aurora B, CGB, and F-actin. Dotted rectangle represented regions that are shown with higher magnification. DAPI, grey, DNA. Scale bars, 50 μm.

Accompanied by an alteration of the nuclear architecture, the term associated with “positive regulation of cell proliferation” was decreased during implantation (Figure 3A). To investigate the proliferation of trophoblast cells in more detail, we first performed cell cycle analysis in all trophoblast clusters. The proportions of the S phase and G2/M phase in TE, pCTB, and pFCC were gradually reduced, indicating that TE and pCTB maintained high proliferative ability; however, pFCC started to lose the proliferative ability (Figure 5A). Next, we checked the cell cycle-associated genes in trophoblast cells. The proliferating marker: *PCNA* and *MKI67* were also downregulated during syncytialization (Figure 5B).

At the end of mitosis, the cytoplasmic bridge joining the daughter cells is checked by the chromosomal passenger complex (CPC) component Aurora B kinase to ensure that chromatin bridges have been resolved and are ready to depart (Mierzwa and Gerlich, 2014). To further confirm cell cycle was arrested in pSTB, we cultured human embryos and immunostained them for cytoplasmic bridge marker, encoded by *AURKB*, in human embryos. It was shown that there were two “cytoplasmic bridges” in TE of day 6 human embryos, which were confirmed by co-immunostained α-tubulin in human embryos (Figure 5C; Supplementary Figure 3A). However, the “cytoplasmic bridges” disappeared in day 10 human embryos (Figures 5C,D; Supplementary Figures 3A,B). These results revealed that primary syncytialization was accompanied by cell cycle arrest and loss of cytoplasmic bridges. Next, to gain insight into if “cytoplasmic bridge” is missing in STB differentiated from hTSCs, we stained Aurora B, CGB, and F-actin in hTSCs and STB cultured for 5 days. The Aurora B^+^ “cytoplasmic bridge” disappeared in STB, similar to the expression pattern in the pSTB in post-implantation human embryos (Figure 5E).

To further explore if cytoplasmic bridges exist in pSTB of human embryos, we immunostained the Day 6 and Day 10 embryos cultured *in vitro* for the specific cytoplasmic bridging marker, Anillin, and pSTB marker, CGB. It was shown that Anillin was enriched in TE cells of day 6 human embryos (Supplementary Figure 4A) and was missed in pSTB of day 10 human embryos cultured *in vitro* (Supplementary Figure 4B). We also co-immunostained the Anillin with Aurora B in day 6 human embryos. It was shown that Anillin could be expressed in Aurora B^+^ location (Supplementary Figure 5A). To better illustrate this situation, we then checked the expression of cytoplasmic bridging markers, such as *KIF4A*, *CENPE*, *PLK1*, and *ANLN* (Hu et al., 2011; Kechad et al., 2012) in the scRNA-seq dataset. It was shown that these markers were decreased in pSTB (Supplementary Figure 4C). Taken together, we concluded that cytoplasmic bridges were lost in pSTB of human embryos.

To obtain the primary syncytialization mechanism in trophoblast cells of human embryos, we used SCENIC analysis in the scRNA-seq dataset and analyzed the top-ranked TFs that regulate primary syncytialization. New potential TFs were elucidated, such as *TCF7L1*, *OVOL2*, *E2F4*, and *BCLAF1* (Supplementary Figures 6A,B). To identify the fusion-associated TFs, we found that *SNAI1*, *BATF*, *KLF10*, and *ZBTB7B* were highly enriched in pFCC (Supplementary Figures 6A,B). It was shown Aurora B induces epithelial-mesenchymal transition by stabilizing Snail1 to promote basal-like breast cancer metastasis (Zhang et al., 2020). This may provide some clues to reveal the mechanism of primary syncytialization. Taken together, these results demonstrated that proliferation of trophoblast decreases with syncytialization during implantation. Moreover, we found some TFs in pFCC, which may offer a new clue to reveal the mechanism of primary syncytialization.

### pSTB Is Related to Cellular Aging

The placental STB has been identified as the major site of the synthesis and secretion of multiple hormones (Barrera et al., 2007; Handschuh et al., 2007; Guibourdenche et al., 2009). To study the function of pSTB in more detail, we analyzed the expressions of the top 100 polypeptide hormone genes that have been recognized as being produced by human placental trophoblasts based on Gene Expression Omnibus (GEO) and previous reports (Guibourdenche et al., 2009; Liu et al., 2018). As expected, 39 polypeptide hormone genes were detected in pSTB. Among them, *CGA*, *PGF*, *INSIG2*, *LHB*, and several *PSG* and *CGB* genes were highly expressed in pSTB2 (Supplementary Figure 7). This result indicated that pSTB during implantation also secreted multiple hormones.

**FIGURE 6:**
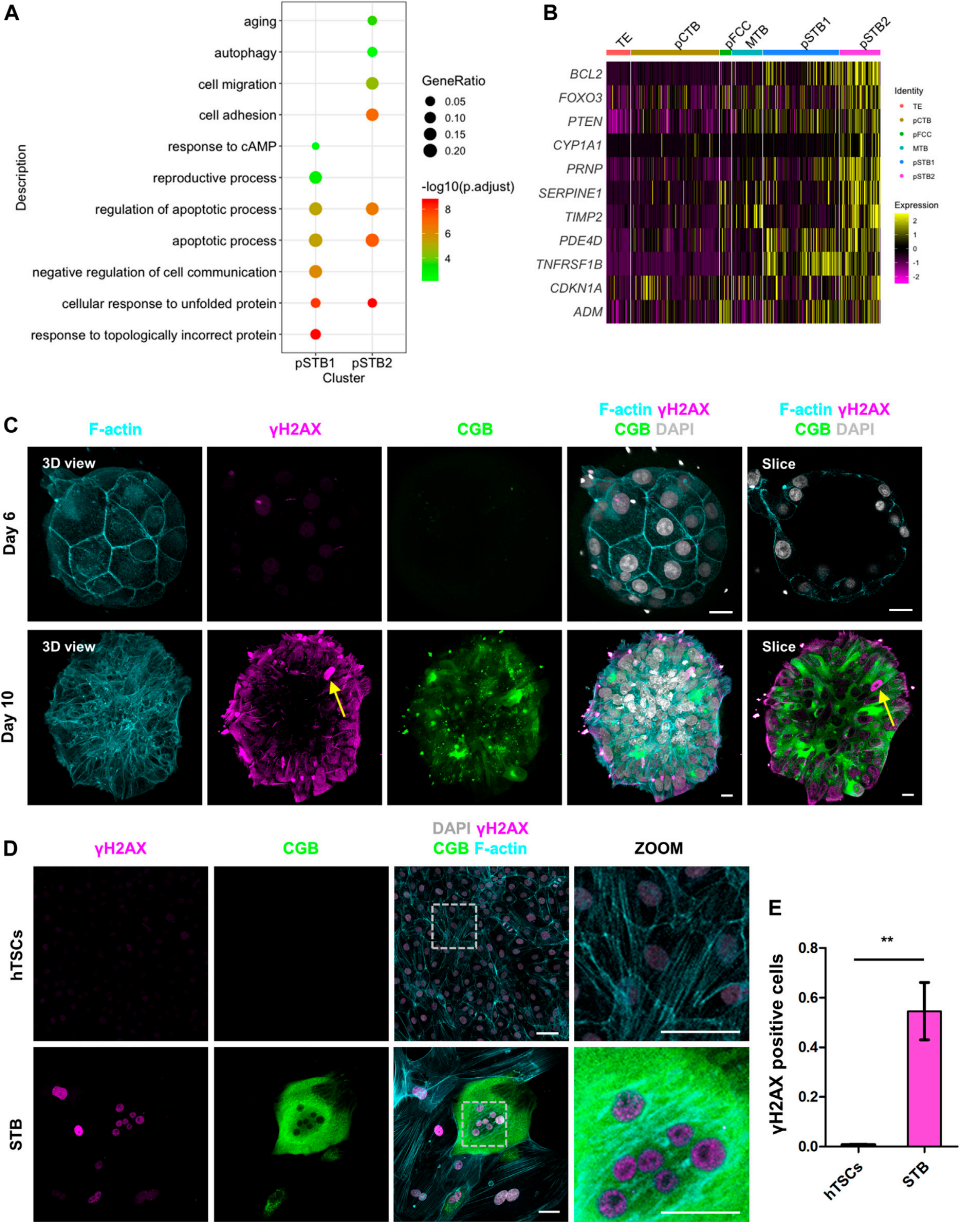
Primitive syncytiotrophoblast is related to cellular aging. **(A)** Dot plots showing enriched GO terms and *p*-value of distinct clusters of trophoblast cells. **(B)** Heat map of indicated genes in distinct clusters of trophoblast cells. The color key from purple to yellow indicates low to high gene expression levels, respectively. **(C)** Immunostaining of day 6 and day 8 human embryos (*n* = 3 and *n* = 3) cultured *in vitro* for F-actin, γH2AX, and CGB. DAPI, grey, DNA. Scale bars, 20 μm. **(D)** Immunostaining of hTSCs and STB for γH2AX, CGB, and F-actin. Dotted rectangle represented regions that are shown with higher magnification. DAPI, grey, DNA. Scale bars, 50 μm. **(E)** Quantification of the numbers of γH2AX positive nuclei in hTSCs and STB. Data are shown as mean ± SEM. *n* = 10 fields of view per group. Unpaired two-tailed Student’s *t*-test, **p < 0.01.

Nuclei deformation and cell cycle arrest are important indications of cellular aging (Kuilman et al., 2010). To determine the relationship between cellular aging and the nuclear morphogenetic changes during primary syncytialization, we performed GO analysis in pSTB1 and pSTB2. In addition to the secretion property of the pSTB, the GO terms enriched in these cells were associated with “cell adhesion” and “response to cAMP”. We also observed that the functions of genes specific for pSTB1 and pSTB2 were mainly related to “apoptotic process” and “autophagy” (Figure 6A). Intriguingly, we identified “aging” enriched in pSTB2 (Figure 6A). At the same time, we noticed that pSTB2 was also enriched for aging-associated genes, such as *BCL2*, *FOXO3*, and PTEN (Figure 6B). Persistent activation of a DNA damage response (DDR) is important for the establishment and maintenance of cellular aging (Bartkova et al., 2006). To detect whether cellular aging occurred in pSTB of human embryos, we cultured human embryos and immunostained them for γH2AX (a marker of DNA damage response), CGB, and F-actin in human embryos. Limited γH2AX foci were detected in TE cells of day 6 human embryos. However, CGB^+^ pSTB contained γH2AX foci (Figure 6C; Supplementary Figure 8A). These results suggested that the increased γH2AX in the pSTB of human embryos might be related to cellular aging. Downregulated levels of LaminB1 have become a common marker of cellular aging. We then checked the expression of LMNB1 in trophoblast cells. It was revealed that the expression levels of LMNB1 were downregulated in pSTB of human embryos (Supplementary Figure 9A). These results suggested that the pSTB in human embryos was related to cellular aging.

We next sought to determine whether cellular aging was also induced during hTSCs syncytialization. We cultured hTSCs in hTSCs medium and STB medium for 6 days and immunostained the cells for γH2AX and CGB. It was shown that compare with hTSC, the CGB^+^ STB expressed higher γH2AX (Figure 6D). We further compared the numbers of γH2AX foci-containing cells in STB and hTSCs. The number of these foci-containing cells was higher in the STB than in hTSCs (Figure 6E).

To illustrate STB was accompanied by cellular aging, we also immunostained STB cultured *in vitro* for Lamin B1; which is also a marker for cellular aging and CGB. Compared with CGB- cells, decreased levels of the Lamin B1 in CGB^+^ STB were detected (Supplementary Figures 9B–D). We stained hTSCs and STB, which differentiated from hTSCs, for senescence-associated-β-galactosidase (SA-β-gal). Increased levels of SA-β-gal activity in STB compared with the one in hTSCs were detected (Supplementary Figure 9E). Taken together, we confirmed that STB was accompanied by cellular aging. These results suggested that the pSTB in human embryos and STB induced by hTSCs were related to cellular aging.

## Discussion

Implantation of blastocysts is vital for mammalian development (Koot et al., 2012, Miller et al., 2012, Norwitz et al., 2001a, b). Mouse trophoblast development in the peri-implantation stage is characterized by highly dynamic gene expressions and cellularly heterogeneous (Rossant and Cross 2001; Cockburn and Rossant 2010). However, trophoblast heterogeneity and the underlying mechanism during implantation, which are pivotal for differentiation in human embryos, remain largely unknown. In this study, we identified the cytoskeleton transition of trophoblast cells based on the scRNA-seq of human embryos. Then, we characterized nuclear deformation and cell cycle arrest during implantation based on immunofluorescence. Altogether, this study enhanced our knowledge of human early trophoblast lineage specification. Moreover, we found syncytialization of hTSCs could also mimic the transition of the cytoskeleton, which could be used as a proper *in vitro* model for future mechanistic study during implantation.

During implantation, TE cells differentiate into trophoblast cells on day 8 post-conception (James et al., 2012; Boss et al., 2018; Turco and Moffett 2019). Studies on the genetic basis of trophoblast differentiation have been largely restricted to mice (Knofler et al., 2001; Simmons and Cross 2005; Kidder and Palmer 2010; Latos and Hemberger 2014; Hemberger et al., 2020). Transcriptional or morphological cytoskeleton dynamics have been shown during implantation in mice (Kidder and Palmer 2010; Wallingford et al., 2013). However, the transcriptional and morphological cytoskeleton dynamics during human embryo implantation have not been reported. Our analysis revealed the reduced expression dynamics of cytoskeleton-associated genes. First, we identified that genes encoding the cytoskeleton, such as *GJA1*, *TUBB4A*, and *ACTB* decreased during implantation (Figure 3B). Combined with transcriptional results, we demonstrated that post-implantation pSTB exhibited obvious nuclear malformations. The “cytoplasmic bridge” also vanished during implantation, suggesting the cell cycle was arrested. These are important indicators of cellular aging. Surprisingly, we also enriched some aging-associated genes in pSTB. The gene dynamics and morphogenesis during human embryo implantation may help to broaden our understanding of implantation.

Primary syncytialization is an essential event during embryo implantation (Turco and Moffett 2019). The distinguishing characteristic of pSTB at this stage is the presence of numerous irregular, slit-like lacunae within the cytoplasm of the STB in human embryos *in vivo* (Carnegie stage 5b-embryo, #8004) and human embryos cultured *in vitro* (Supplementary Figure 10). However, lacunae structure was not found in STB at later pregnancy (for example, 8-week placental villi). The STB has also been considered as the major site of the synthesis and secretion of hormones. STB_8W expressed 60 hormone genes, including those of well-documented gene families, such as *CGB* and *PSG* (Liu et al., 2018). However, some of these genes were not expressed in pSTB. For instance, *PAPPA*, *KISS1*, and *CSH1* were significantly downregulated in the pSTB during implantation (Supplementary Figure 7). Thus, the ability of STB to secrete hormones gradually increases over trophoblast development, and STB gradually matured. The temporal changes in the expression of these polypeptide hormone genes may reflect different roles for STB during implantation and placentation. It gives us a hint that compared with STB during placentation, pSTB may be immature. However, it needs to be supported by experimental data basing on a reliable investigating model.

The underlying mechanism of primary syncytialization has not been elucidated. *ERVW-1* (*Syncytin 1*) and *ERVFRD-1* (*Syncytin 2*) are usually associated with syncytialization in placental villi (Mi et al., 2000; Blaise et al., 2003; Frendo et al., 2003; Langbein et al., 2008). *Syncytin2* is a signature for placental fusion-competent cells. Only CTBs that have exited the cell cycle and have highly expressed *Syncytin2* can fuse (Liu et al., 2018; Lu et al., 2018). Leveraging our scRNA-seq of human embryos, we found pFCC enriched in “cell migration” function (Figure 3A). Several TFs in each trophoblast subtype have also been revealed. For instance, we found that *SNAI1*, *BATF*, *KLF10*, and *ZBTB7B* were highly enriched in pFCC (Supplementary Figures 6A,B). Further studies are urgently needed to address the indisputable roles of these TFs in primary syncytialization.

Studies on primary syncytialization during implantation have been largely restricted to sections of the Carnegie Collection (Hertig et al., 1956). Prior studies have noted the urgent need for an *in vitro* model to mimic this situation during implantation. Commonly *in vitro* models are choriocarcinoma cell-derived spheroids (Rothbauer et al., 2017) and human embryonic stem cells (hESCs) derived trophoblast-like cells (Xu et al., 2002; Yue et al., 2020). However, choriocarcinoma cells possess cancer features and the transcriptome is different from those of primary trophoblast cells. Trophoblast-like cells derived from hESCs resemble human trophectoderm during implantation but not subsequent syncytiotrophoblast. Nowadays, the system of culturing human embryos to post-implantation stage is also established. However, owing to the limited number of human embryos available and ethical issues for genetic manipulation, it is necessary to obtain a reliable model to investigate trophoblast development during implantation. Recent developments in hTSCs that are cultured atop a two-dimensional surface have fulfilled the need for trophoblast specification (Okae et al., 2018). Whether hTSCs could be used for investigating trophoblast differentiation during implantation remains an open question. In this study, syncytialization of hTSCs has also been proved to remodel some morphological features of primary syncytialization, suggesting hTSCs could be a platform to investigate the morphogenesis of trophoblast differentiation during implantation.

In conclusion, our study provides valuable resources for deciphering comprehensive gene expression landscapes of heterogeneous trophoblast cells and morphogenesis initiated upon embryo implantation. These findings are potentially valuable in advancing not only our current understanding of implant progression but also comprehension of early pregnancy loss in humans.

## Data Availability

The raw data supporting the conclusions of this article will be made available by the authors, without undue reservation.
