# Supplementary material for: A Single-Cell Characterization of Human Post-implantation Embryos Cultured In Vitro Delineates Morphogenesis in Primary Syncytialization
Source: Front Cell Dev Biol. 2022 Jun 15;10:835445. doi: 10.3389/fcell.2022.835445 (PMC9240912; doi:10.3389/fcell.2022.835445)
Supplement: Supplementary file 1 [file DataSheet1.pdf]

Supplementary Materials for

A single-cell characterization of human post-implantation embryos cultured *in vitro* delineates morphogenesis in primary syncytialization

This PDF file includes:
Supplementary Figure 1 to 4
Supplementary Text
Supplemental Code
Supplementary Table 1 to 2

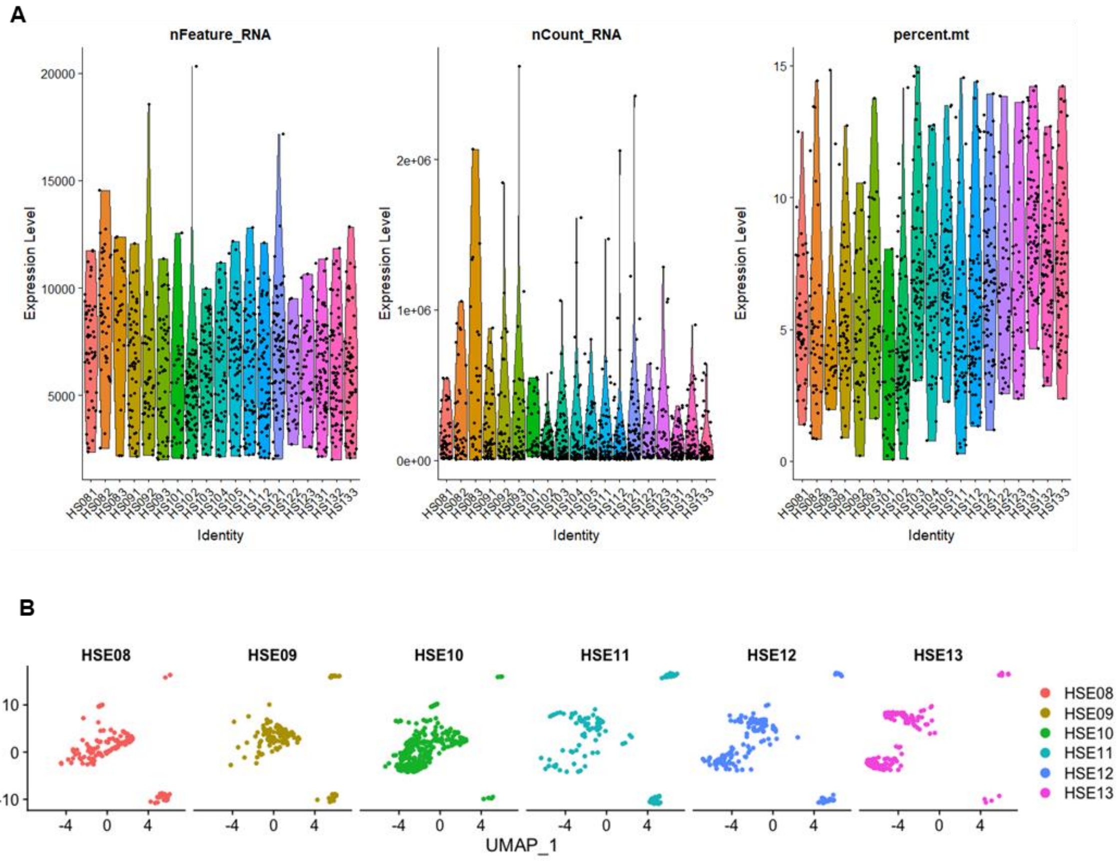

Supplementary Figure 1. Single-cell RNA-seq of human embryos cultured *in vitro*

A. Violin plots showing the numbers of genes expressing more than 0 (left), total expression of all genes (middle), and percentage of mitochondrial gene expression (right) detected in every single cell.

B. The number of cells per embryonic day (day 8-day 13) retained after quality filtering.

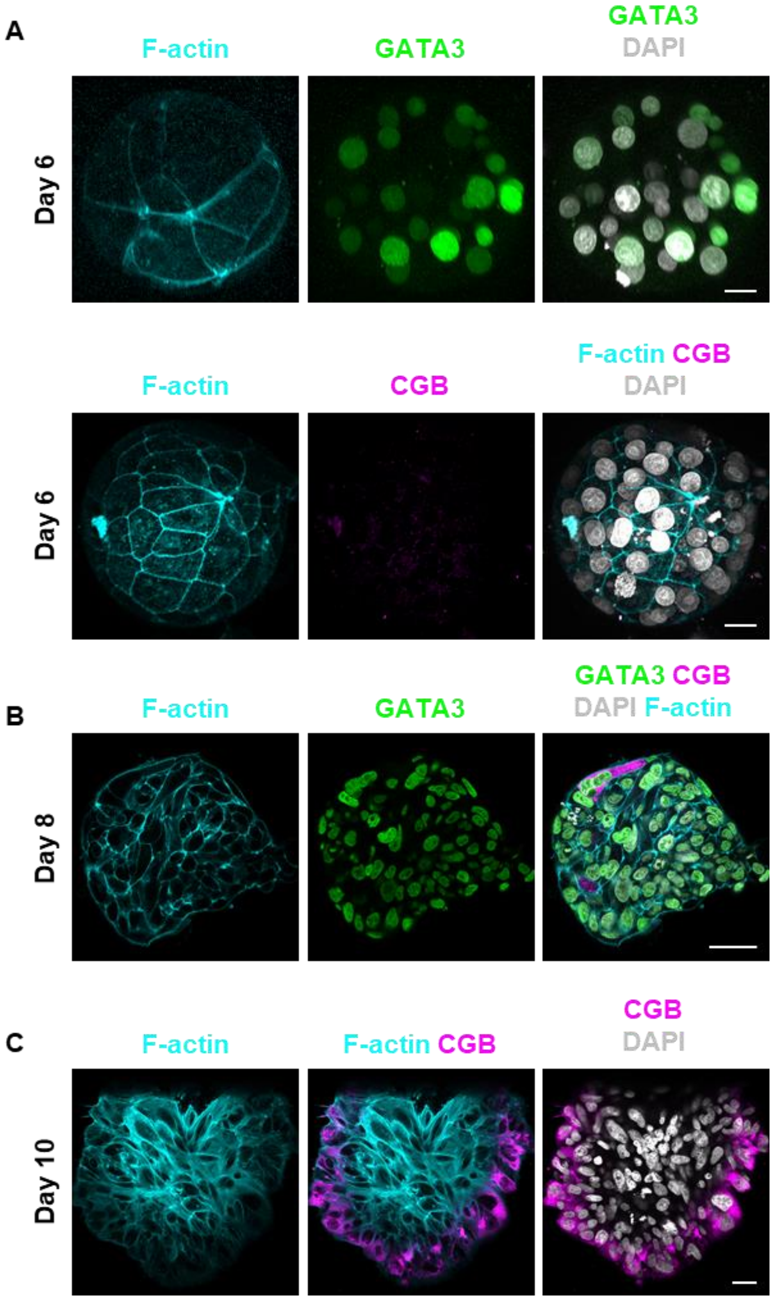

Supplementary Figure 2. Immunostaining of human embryos for TE and trophoblast lineage markers.

A. Immunostaining of day 6 human embryos for F-actin, GATA3, and CGB. DAPI, grey, DNA. Scale bars, 20 µm.

B. Immunostaining of day 8 human embryos for F-actin, GATA3, and CGB. DAPI, grey, DNA. Scale bar, 50 µm.

C. Immunostaining of day 10 human embryos for F-actin and CGB. DAPI, grey, DNA. Scale bar, 50 µm.

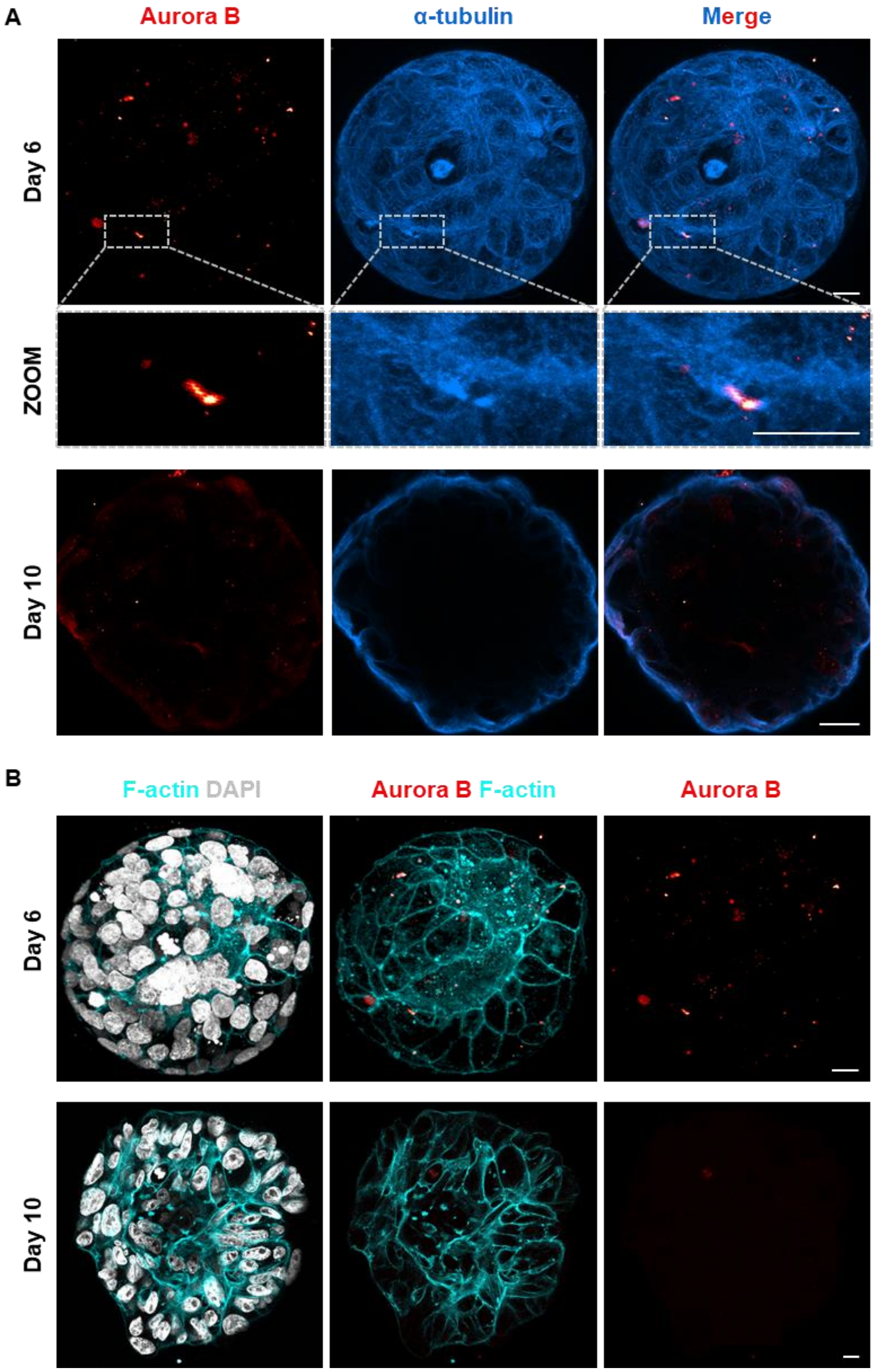

Supplementary Figure 3. Verification of 'cytoplasmic bridge' in human embryos.

A. Immunostaining of day 6 and day 10 human embryos (n=2 and n=2) cultured *in vitro* for α-tubulin and Aurora B. Dotted rectangle represented regions that are shown with higher magnification. Scale bars, 20 μm.

B. Immunostaining of day 6 and day 10 human embryos (n=3 and n=3) cultured *in vitro* for F-actin and Aurora B. DAPI, grey, DNA. Scale bars, 20 μm.

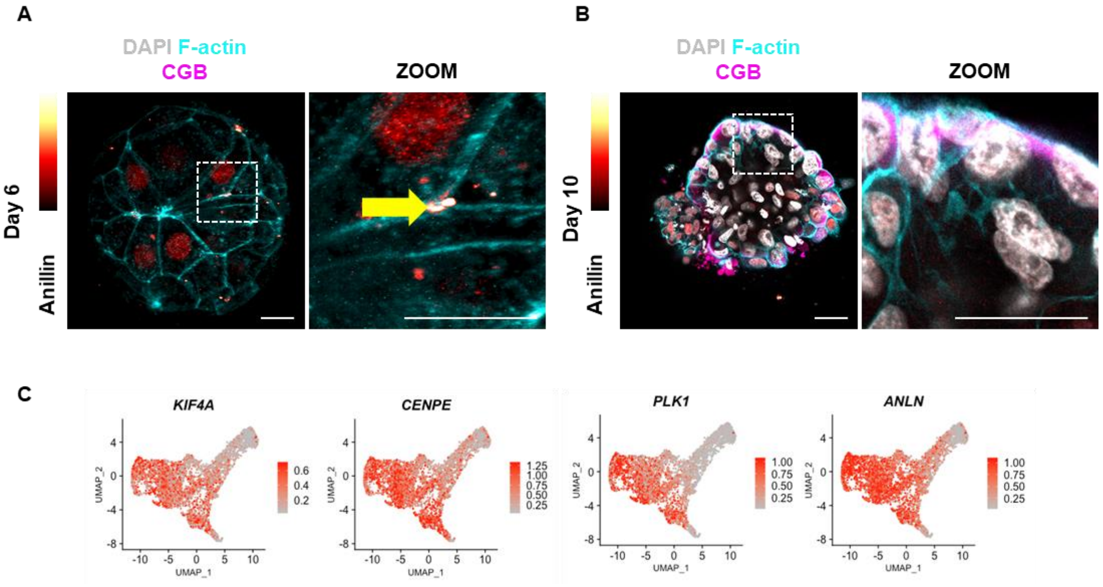

Supplementary Figure 4. Loss of ‘cytoplasmic bridge’ in pSTB.

A. Immunostaining of day 6 human embryos for F-actin, CGB, and Anillin. DAPI, grey, DNA. Dotted rectangle represented magnified area. Yellow arrow indicated ‘cytoplasmic bridge’. Scale bars, 20 µm.

B. Immunostaining of day 10 human embryos for F-actin, CGB, and Anillin. DAPI, grey, DNA. Dotted rectangle represented magnified area. Scale bars, 50 µm.

C. UMAP plots showing the indicated genes in trophoblast cells. Color key from grey to red indicates relative expression levels from low to high, respectively.

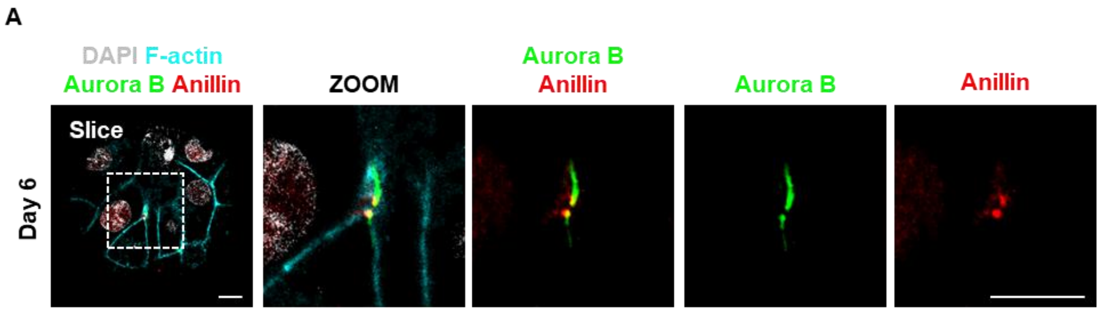

Supplementary Figure 5. Co-immunostaining of 'cytoplasmic bridge' in human embryos.

A. Immunostaining of day 6 human embryos for F-actin, Aurora B, and Anillin. DAPI, grey, DNA. Dotted rectangle represented magnified area. Scale bars, 10 μm.

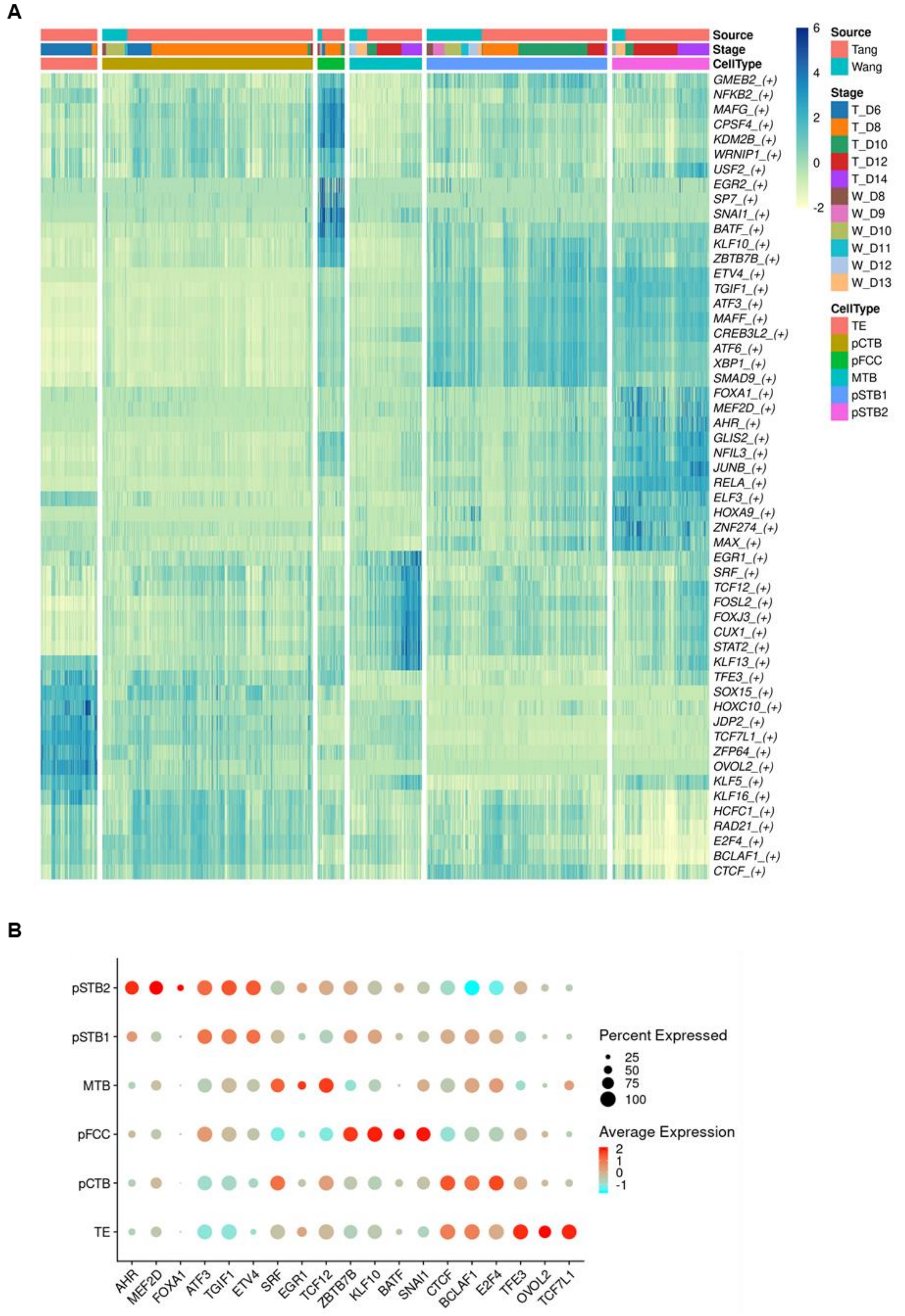

Supplementary Figure 6. Transcription factors expressed in the trophoblast cell subtypes.

A. Heat map of transcription factors expressed in the trophoblast cell subtypes.

B. Dot plot showing newly identified TFs in pFCC. The color key from cyan to red indicates low to high gene expression levels, respectively.

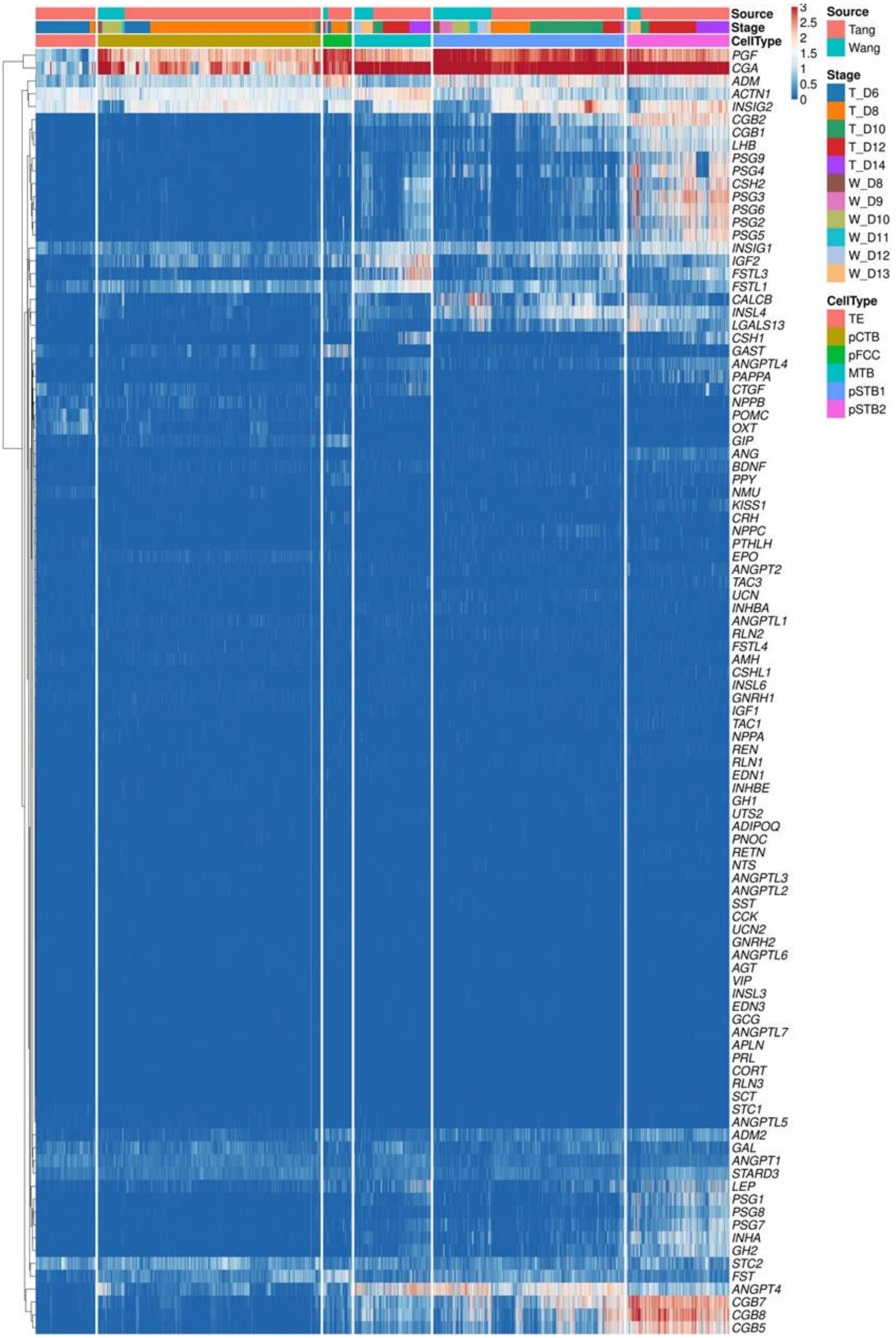

Supplementary Figure 7. Heatmap of polypeptide hormone genes expressed in subtypes of trophoblast cells.

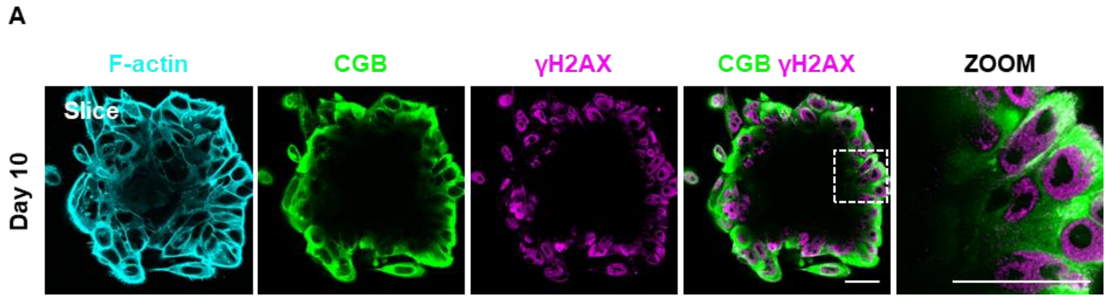

Supplementary Figure 8. Expression of cellular aging-related genes in human embryos.

A. Immunostaining of day 10 human embryos for F-actin, CGB, and γH2AX. DAPI, grey, DNA. Dotted rectangle represented magnified area. Scale bars, 50 μm.

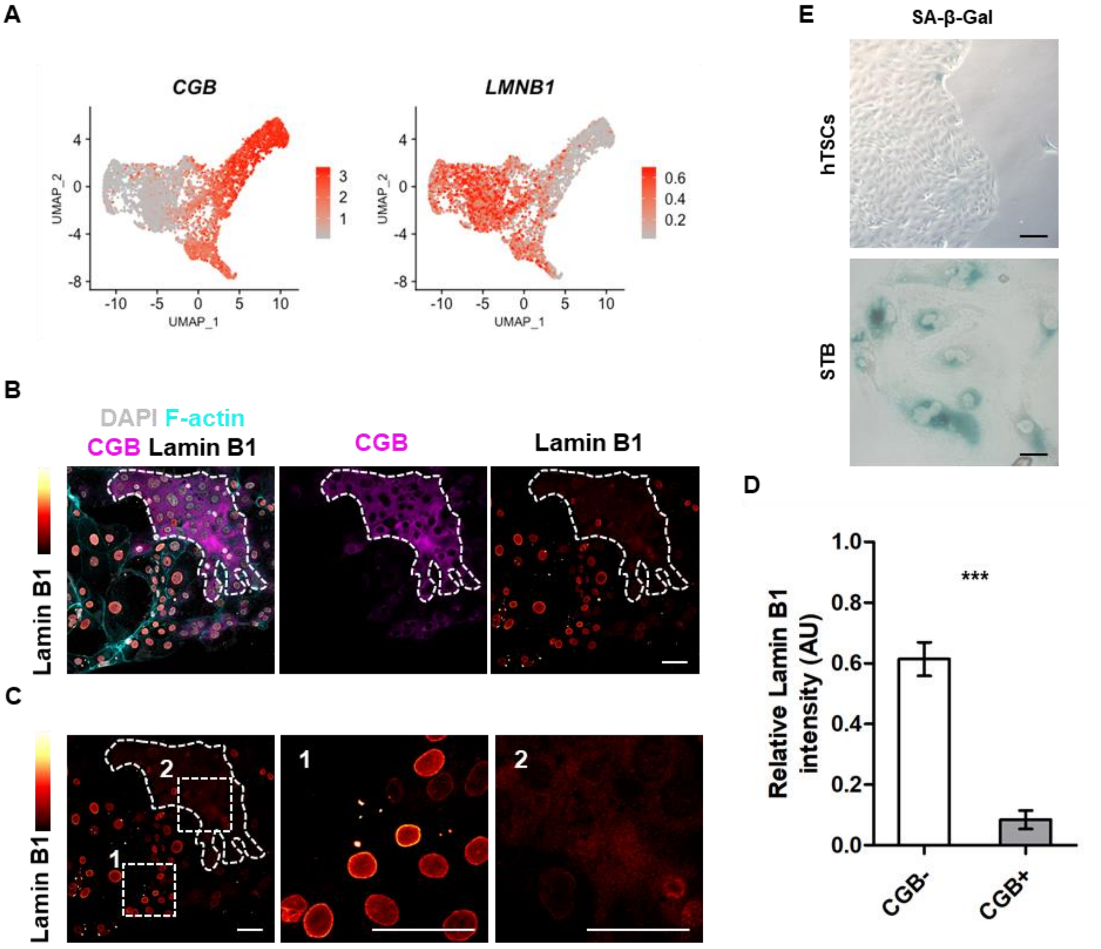

Supplementary Figure 9. Expression of cellular aging-related genes in human embryos and STB differentiated from hTSCs.

A. UMAP plots showing the indicated genes in trophoblast cells. Color key from grey to red indicates relative expression levels from low to high, respectively.

B. Immunostaining of STB for F-actin, CGB, and Lamin B1. DAPI, grey, DNA. Scale bar, 50 μm.

C. Immunostaining of STB for Lamin B1. DAPI, grey, DNA. Dotted line represented CGB^{+}STB. Dotted rectangle represented magnified area. Scale bars, 50 μm.

D. Relative Lamin B1 intensity of images in B. Data are presented as the mean ± s.e.m.. ***$P < 0.001$.

E. SA-β-gal staining of hTSCs and STB at passage 20. Scale bars, 50 μm.

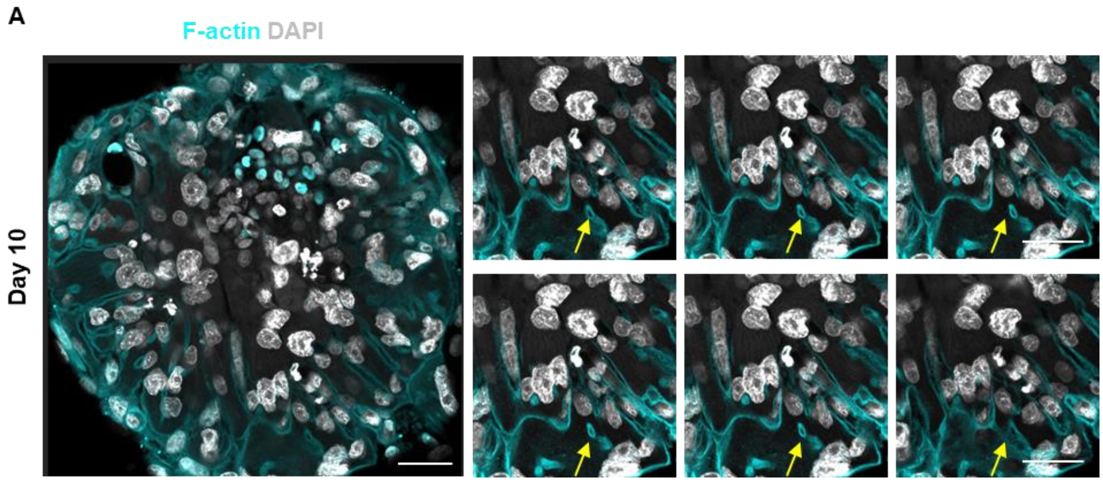

Supplementary Figure 10. Immunostaining of F-actin in human embryos.
A. Immunostaining of day 10 human embryos for F-actin. DAPI, grey, DNA. Arrows represented 'lacunae' in pSTB of human embryos. Scale bars, 50 μm.

Supplementary Text

Competing financial interests

The authors declare no competing financial interests.

Materials & Correspondence

Correspondence and material requests should be addressed to Hongmei Wang.

Supplemental Code: eryxoksanzglhqj

Supplementary Table 1. Antibodies

Name	Manufacturer name	Code number	Dilution	Lot	Polyclonal or Monoclonal
**Phalloidin**	YEASEN	40735ES75	1:200	F1001230	N/A
**CGB**	Golden Bridge Biotechnology	ZM-0134	1:200	N/A	monoclonal
**CGB**	Abcam	ab53087	1:200	899357	polyclonal
**GATA6**	RD	AF1700	1:200	N/A	polyclonal
**OCT4A**	Cell Signaling Technology	2840P	1:200	9	monoclonal
**Lamin A**	Abcam	ab26300	1:200	GR3222248-1	polyclonal
**γH2AX**	Abcam	ab2893	1:200	GR3270858-1	polyclonal
**Aurora B**	Abcam	ab2254	1:200	GR3210135-1	polyclonal
**α-tubulin**	YEASEN	30304ES40	1:200	T16546	monoclonal
**Anillin**	Abcam	ab211872	1:100	GR3271951-21	monoclonal
**Lamin B1**	Zen BioScience	201169	1:100	EE1124	monoclonal
**GATA3**	Cell Signaling Technology	5852S	1:200	5	monoclonal

Supplementary Table 2. Number of human embryos for scRNA-seq

Time	Number of human embryos
Day 8	2
Day 9	3
Day 10	3
Day 11	2
Day 12	1
Day 13	1
